# Innovate and Stockpile: Respiratory Protection for Essential Workers in a Catastrophic Pandemic

**DOI:** 10.1089/hs.2022.0126

**Published:** 2023-07-18

**Authors:** Nadia X. Montazeri, Jonas B. Sandbrink

**Affiliations:** Nadia X. Montazeri, MD, is a Research Affiliate, Center for International Security and Cooperation, Stanford University, Stanford, CA.; Jonas B. Sandbrink is a Doctoral Researcher, Nuffield Department of Medicine, University of Oxford, Oxford, UK.

**Keywords:** COVID-19, Public health preparedness/response, Personal protective equipment, Hospital preparedness/response, Global catastrophic biological risk

Suitable respiratory protective equipment (RPE) is crucial for responding to highly deadly airborne viruses, or pathogens that could cause a Global Catastrophic Biological Risk (GCBR). GCBRs are defined as worst-case biological events that could threaten the stability of society.^1^ Almost by definition, a GCBR event will be more lethal than the COVID-19 pandemic. For example, if COVID-19 had exhibited a similar level of severe disease as SARS, which had a case fatality ratio (CFR) of 11%, the resulting pandemic might have constituted a GCBR. Furthermore, case fatality ratios may be even higher for respiratory viruses, as demonstrated by the Middle East respiratory syndrome (CFR of 32.7%)^2^ and avian influenza (CFR of 52.6%)^3^. High levels of lethality and transmissibility may occur especially for engineered agents. While we cannot predict the exact properties of a GCBR-level pathogen, airborne transmission is most likely to cause exponential spread. Physical countermeasures such as RPE can help protect people from many different respiratory viruses and thus can be effectively prepared in advance. In this commentary, we examine the importance of RPE for GCBR preparedness and examine favorable properties of possible products and deployment strategies. The Table outlines challenges, implications, and recommendations for respiratory protection in a GCBR scenario.

Alongside gloves, gowns, and goggles, RPE is a component of personal protective equipment (PPE). The most common RPE is the filtering facepiece respirator (FFR), such as the N95 and N99 respirators in the United States and FFP2 and FFP3 respirators in Europe. During the COVID-19 pandemic, medical masks and barrier face coverings were used as respiratory protection to compensate for respirator shortages. Reusable equipment was applied at varying amounts^4^ but played a minor role compared with disposable equipment. Examples of reusable RPE include elastomeric half-mask respirators, powered air-purifying respirators, and respiratory equipment for chemical, biological, radiological, and nuclear (CBRN) incidents. At the moment, it is unknown how countries will adapt their pandemic preparedness plans, including respiratory PPE strategies. Such updates should acknowledge the risks posed by more dangerous pathogens.

**Table:** Table. Characteristics of a Global Catastrophic Biological Risk Compared With the COVID-19 Pandemic and Subsequent Implications for Respiratory Protection

Challenge	Implication	Recommendation
GCBR pathogen is more lethal	N95-level FFR protection could be insufficient even if fitted perfectly	Keep developing products beyond N95-level effectiveness and include them in stockpiles
	Less tolerance for remaining infection risk when wearing imperfect RPE	
	FFR protection level is insufficient without proper fit	Prioritize products that do not require fit testing and/or incentivize proper use by design
GCBR pathogen is scarier	Essential workers need to trust their RPE	Foster trust in RPE by designing products to give a feeling of safety and protection
	Essential workers need *justified* trust in RPE: Tangible evidence of effectiveness	Create measures beyond filtration efficiency that model real-world effectiveness better, such as human challenge trials with airborne pathogens that are safe (no severe symptoms, not transmissible between humans) Analyze nuanced evidence for effectiveness considering imperfect and ideal use, similar to the range of the Pearl Index for contraception
GCBR pathogen spreads faster	Products need to be ready to ship from day 1	Focus on the importance of stockpiling the right product
	Disrupted supply chains	Stockpile reusable products in case scaling up manufacturing quickly is not possible
The ideal RPE product for a GCBR does not exist yet	Incentivize innovation	Regulatory: Create testing protocols specific for high-end pandemic respiratory protection beyond filtration efficiency Financial: Set up prizes for innovative designs and advanced market commitments that bring products all the way to the shelf

Abbreviations: FFR, filtering facepiece respirator; GCBR, global catastrophic biological risk; RPE, respiratory protective equipment.

## Protecting Essential Workers

In a GCBR scenario, RPE could play a key role in keeping essential workers in their jobs and thus, keeping society running. In a pandemic that is highly lethal across all age groups, those who do not urgently need to leave the house will likely choose to stay home. This was observed for vulnerable populations during COVID-19. For example, cystic fibrosis patients in the United Kingdom reported adhering to the strict government-recommended shielding measures.^5^ Similarly, a survey in Spain found that people who perceived themselves as vulnerable were more likely to adhere to protection measures.^6^ However, even in the face of a GCBR, certain individuals will have to leave their houses to keep society from collapsing. Because society relies on a division of labor around the most basic of human needs, a small number of individuals will be particularly essential to society's survival. These essential workers include not just nurses and doctors but also those working in critical infrastructure, such as food and water supply, waste management, and energy production. Critical infrastructure workers need protective equipment to not only prevent infection and death, but also to encourage them to keep doing their indispensable work.

Existing approaches to respiratory protection do not include essential workers outside of healthcare. Healthcare professionals are the only group with a chance of getting regularly fit-tested and trained in RPE usage. Most essential sectors do not have a respiratory protection program, and therefore cannot provide their workers with relevant training or equipment.

An important aspect of RPE is that it enables individual resilience and agency, which helps protect key workers. In contrast to other nonpharmaceutical interventions like limitations of social gatherings, any individual can use RPE to protect themselves without relying on quick government action or others' compliance with protection measures such as social distancing or lockdowns. This ability to act individually can assist in preventing society from collapsing, as it enables subsets of more careful populations to protect themselves and might also reduce societal friction. For RPE to provide maximum safety to those risking their health, its physical properties and deployment strategies must be suitable for a GCBR setting.

## Properties of Better Products

A highly lethal pathogen demands a highly effective countermeasure because of increased health risks to individuals. The main physical requirement is the ability of a device to protect its wearer from infection without interfering with their work. Fortunately, medical masks protected well against early SARS-CoV-2 variants, and even improvised protection devices like cloth masks contributed to a slower spread.^7,8^ Against a GCBR-level pathogen, it is much less likely that homemade masks could offer sufficient protection to make people feel comfortable enough to leave their homes. We therefore recommend diversifying pandemic preparedness efforts to include high-end protective equipment.

It is unclear if commonly used N95-style FFRs offer enough protection for a highly lethal, highly infectious pathogen. Assuming a perfect fit, N95-style FFRs such as KN95s, KF94s, and FFP2s reduce particulate matter of the most penetrating particle size (MPPS, 0.3 microns on average) to 5% or 6%.^9^ Depending on the minimum infectious dose of the pathogen and the concentration and size of virus particles in the air, this filtration may or may not suffice for an extremely infectious pathogen. We therefore recommend more research on the protection these devices provide in cases where a small number of particles might suffice to cause an infection.

A key problem of existing RPE is that measured effectiveness is dependent on a good fit. To avoid leakage, nonpowered air-purifying half-mask respirators (both elastomeric respirators and FFRs) require a seal around the chin, cheeks, and nose bridge. The right product for an individual's facial features and the correct donning technique are usually evaluated during a fit test. We expect that just-in-time fit-testing is not a realistic option in a GCBR scenario because nonhealthcare workers are excluded from regular respiratory protection programs and will require well-fitted RPE. A GCBR moves too fast to fit-test everyone who needs protection.^10^ The US Centers for Disease Control and Prevention estimates that a facility with 1 experienced operator and appropriate logistics support could fit-test up to 400 people in an 8-hour workday. While this rate is already ambitious in a large hospital with smooth operations, it is unrealistic to apply it in rural locations that do not have fit-testing equipment or an experienced operator onsite already. Another design problem with common negative-pressure devices is that they incentivize incorrect fit because leakage eases breathing. In addition, KN95-style flatfold respirators with ear loops became popular, especially among the public. Replacing head straps with earloops, however, decreases fit significantly, as investigated by Niu et al.^11^

High-end equipment already on the market is CBRN-type gear, which is reusable. CBRN-level respirators would likely be effective in protecting against a GCBR-level pathogen. However, CBRN-level respirators did not gain widespread popularity during the COVID-19 pandemic, although a study with self-selected participants in an Australian hospital noted that staff preferred the CBRN respirator over their usually worn N95 respirator, donning was quicker, and short training was sufficient.^12^ The downsides of the CBRN respirator are that it is incompatible with eyeglasses and has limited source control, potentially allowing pathogens from an infected wearer to escape through the exhalation valve and infect others.^13^ We recommend creating more options for highly effective products, including products that can be worn with glasses and that filter exhaled air. The ideal product provides universal fit without fit testing or training and incentivizes correct donning, for example, by integrating an automated feedback mechanism.

In addition to filtration efficiency and proper fit, other factors should be considered when evaluating the physical properties of RPE. When selecting a product to safeguard against a highly lethal pathogen, the focus shifts to prioritizing worker protection and ensuring their sense of security throughout their entire shift. This emphasis on worker safety may lead to compromises in source control, potentially enabling the use of CBRN respirators by nonmilitary essential workers. Additional physical properties that are desirable for future GCBR-level pathogens derive from additional modes of transmission, such as fomite transmission, transmission from aerosols to eye mucus membranes, and other unforeseen hazards. They could be addressed with antimicrobial surfaces and innovative, easy disinfection methods, full-face protection design, and modular design, allowing smooth integration with full-body PPE.

## Social Factors for Effective Use of PPE

The effectiveness of RPE is not solely defined by its physical properties but also by social and psychological factors. In contrast to PPE compliance during the COVID-19 pandemic, compliance during a GCBR event might not be the key challenge because exposure to the pathogen would be associated with very obvious risk to the individual. We would expect different challenges for RPE deployment, such as convincing users that devices are reliably protecting them from death and severe disease. An integrative review assessing 40 surveys and interviews with healthcare workers found that PPE is a relevant factor for increasing willingness to work in response to biological events.^14^ During the COVID-19 pandemic, however, people tolerated suboptimal PPE that had a substantial remaining risk of infection, such as surgical or cloth masks, because the risk of severe disease was low for healthy, young individuals. In contrast, the desire for the best possible protection before risking exposure could be stronger in a GCBR scenario. One facet of providing good PPE to essential workers is convincing them that the products provided will perform well. The solution can be 2-fold: first, communicate clearly about the efficacy and evidence of those products in government messaging; and second, double down on existing products or design new ones that make the wearer feel particularly well-protected. This approach is supported by findings from a survey by Hines et al,^15^ which showed that reusable RPE such as powered air-purifying respirators and elastomerics were more popular among healthcare workers in high-risk scenarios despite lower ratings of comfort. We therefore recommend designing products that invoke trust in their effectiveness, even if trading off against comfort or style, and collecting and communicating scientific evidence that justifies this trust.

## Importance of Stockpiling

Stockpiles of RPE are essential in preparing for the fast spread of a GCBR-level agent. A substantial challenge in the surge of a GCBR will likely be a fast and sudden spread, combined with a disruption to infrastructure. The events of the first weeks will determine whether an outbreak turns into a global catastrophe; hence, fast and consequential action is required.

zJust-in-time manufacturing of gear was sufficient in COVID-19 but will not be fast enough for a GCBR. For instance, a deliberately released agent might spread faster than COVID-19, as it could be released at multiple sites and might even be optimized for transmissibility. We already know that transmissibility beyond SARS-CoV-2 or influenza is biologically possible, as the basic reproduction number of measles is between 12 and 18,^16^ which exceeds even that of the Omicron variant at about 9.5.^17^ The COVID-19 response included scaling up existing production of PPE and creating ad hoc supply chains for disposable products.^18^ This effort was remarkable, but it took valuable time. Next to the speed of manufacturing itself, during a GCBR event, public health authorities will have less capacity to provide iterative updates on which products they recommend for those who have been exposed. Gradually increasing the recommended level of protection would require too many victims. We therefore need to select and stockpile the best products we can before a GCBR event.

Additionally, in the face of a sudden and exceptionally transmissible pathogen, existing manufacturing and transport infrastructure could be heavily disrupted. For example, border closures affect transport, a sudden increase in demand can reveal existing bottlenecks in supply chains (eg, meltblown polypropylene for FFRs), and military conflict could actively destroy physical infrastructure.^19^ Extreme risk events highlight the importance of protected stockpiles, particularly of reusable, durable products, when existing manufacturing infrastructure is affected. Maintaining decentralized manufacturing capacity could further strengthen resilience.

## Developing and Testing New Products: Incentivizing Innovation

The development of new and effective RPE can be stimulated from multiple angles. FFRs, elastomerics, and CBRN-level equipment have been developed against a broad range of threats. Our current RPE options, however, have not been designed to be used by untrained workers in the face of a deadly pandemic threat. Different use cases trade off against each other in product design; hence, excellent pandemic preparedness depends on innovation,^20,21^ which can be encouraged with financial incentives and changes to the regulatory landscape.

Financial incentives can spur innovation, but currently there is limited monetary incentive to optimize for highly effective RPE. COVID-19 prompted many innovative ideas for respirators and masks in the areas of style and practicality,^22^ but none of them so far have been adopted widely.^23^ In the early stages of an outbreak event, competitions such as the Mask Innovation Challenge can crowdsource new ideas.^24,25^ Advanced market commitments such as COVID-19 Vaccines Global Access (COVAX) can enable the necessary research and development investments to move promising prototypes to widespread adoption.^26^ Health preparedness authorities like the US Biomedical Advanced Research and Development Authority (BARDA) and the EU Health Emergency Preparedness and Response Authority (HERA), and global partnerships similar to the Coalition for Epidemic Preparedness Innovations (CEPI) could facilitate and fund testing and iterating of new products.^27^

On the regulatory end, current international (International Organization for Standardization [ISO]) and European (European Committee for Standardization [CEN]) standards do not adequately cover the use case of respiratory protection in a GCBR. European testing protocols, for instance, rely on individuals experienced in wearing respiratory protection. In a GCBR scenario, however, untrained individuals need to make as few errors as possible. If tests with dust, radioactive fallout, and chemical vapor were omitted, future standards would allow for more innovation.^28^ For example, a narrow focus on infection protection could enable the development of RPE based on the inactivation of pathogens through ultraviolet irradiation or nanomaterials.

Finally, RPE testing protocols should model as well as possible real-world effectiveness against a highly transmissible pathogen in a GCBR scenario. First, testing methods that model protection better than filtration of sodium chloride particles or latex spheres should be explored. For example, human challenge trials with safe airborne pathogens that are not transmissible between humans could be developed to create more reliable evidence.^29^ Second, the effectiveness of a given product, even if the product is brilliantly designed, still depends on human factors. Similar to the Pearl Index for contraception, a range indicating effectiveness could capture the differences between ideal RPE use and imperfect use on an ongoing basis.^30^

## Stockpiling Strategy

If measures are taken to foster innovation, we have reason to hope for better RPE products. However, innovation takes time, and improving on current stockpiles of disposable gear is urgent. Like others have suggested, adding elastomeric respirators to stockpiles could be a good interim solution.^31^ As products expire and need to be exchanged, they can be replaced with new products, creating increased demand.

For a well-considered stockpiling strategy, it is essential to know the number of people who require protection. Governmental health and defense agencies should consider how many essential workers need high-end RPE supplies to maintain the absolute minimum critical infrastructure functionality. Ideally, enough effective RPE for all indispensable workers should be stockpiled, so that it can be distributed upon a defined trigger. A small fraction of critical infrastructure workers could also be encouraged to store durable equipment at their homes or in local offices. Advantages of decentralized storage and local supply chains should be considered when setting up stockpiles.

## Conclusion

During the COVID-19 pandemic, lower levels of respiratory protection were sufficient against infection. However, this level of protection is not enough to defend against future pandemics of unprecedented fatality. To keep up the fight, the essential workforce needs excellent RPE that is reliable, durable, and instantly available. To that end, governments need to incentivize innovation and stockpile reusable products.
